# Study of Microstructure and Performance Evaluation of Zr-Sn-Nb Joints by Electron Beam Welding

**DOI:** 10.3390/ma17050980

**Published:** 2024-02-20

**Authors:** Yanli Zhao, Anrui Zhang, Huifang Yue, Houqin Wang, Yong Xin, Yi Zhou

**Affiliations:** 1 Science and Technology on Reactor System Design Technology Laboratory, Nuclear Power Institute of China, Chengdu 610213, China; zyl695662724@163.com (Y.Z.);; 2 National Key Laboratory of Precision Welding & Joining of Materials and Structures, Harbin Institute of Technology, Harbin 150001, China

**Keywords:** Zr-Sn-Nb alloy, electron beam welding, microstructure, joint properties

## Abstract

In this work, Zr-Sn-Nb alloy was joined by electron beam welding (EBW). A defect-free Zr-Sn-Nb joint with sound appearance was obtained. The grains in the weld zone (WZ) and heat-affected zone (HAZ) are significantly coarsened. The columnar grains with a maximum grain size of 0.5 mm are distributed in the upper region of the WZ, while the equiaxed grains are almost located in the bottom region of the WZ. The WZ is mainly composed of the dominant α-Zr, α′-Zr and a few β phases. The grain orientation of WZ and HAZ is uniform, indicating that no obvious preferred orientation existed. Coarse grains and fine acicular α′ phases increase the strength of the joint, but reduce the plasticity and toughness of the joint. The tensile strengths of the joints at room temperature (RT) and 375 °C were 438 MPa and 313 MPa, respectively. The RT impact energy of the joint is 18.5 J, which is only 58.3% of the BM. The high purity of the EBW process and unsignificant grain orientation minimizes damage to the corrosion resistance of Zr-Sn-Nb alloy joints. The corrosion weight gain of the joint specimen and the BM specimen were 12.91 mg/dm^2^ and 12.64 mg/dm^2^, respectively, and the thicknesses of the cross-section corrosion layer were 12–15 μm and 9–12 μm, respectively.

## 1. Introduction

Zirconium alloys have been widely used in nuclear reactors due to their resistance to alkaline water corrosion containing boron and lithium, excellent high-temperature mechanical properties, and low neutron absorption cross-sections [1,2]. The development of nuclear reactors has put forward higher requirements on the service life and availability of zirconium alloy components. Under the premise of ensuring the safety, high efficiency and economy of the reactor, the fuel assembly burnup should be more than 50 GWd/tU. Traditional Zr-4 alloys and improved Zr-4 alloys can no longer meet the current high burnup requirements [3,4]. Consequently, the development of new zirconium alloys is imperative. The production of Zr-Sn-Nb alloys by adding a variety of alloying elements to improve the corrosion resistance, mechanics and other properties of zirconium alloys has become a major trend in the research and development of new zirconium alloys, such as Westinghouse’s Zirlo alloy in the U.S., Russia’s E635 alloy, as well as China’s N18 and N36 alloys [5,6,7].

Welding technology of zirconium alloy is of great significance to improve the manufacturing quality and productivity of zirconium alloy fuel assemblies [8,9]. Zirconium is a chemically active metal, and its welded joints are susceptible to contamination by airborne gases such as oxygen, nitrogen and hydrogen, as well as grain coarsening and the deterioration of joint properties due to the low thermal conductivity of the weld molten pool heat accumulation [9]. For this reason, additional protective measures are required during the zirconium alloy welding process.

When zirconium alloys are welded by gas-shielded arc welding, measures such as the addition of trailing shields and backing shields, i.e., the extension of the shielding gas supply, are required to avoid joint contamination [8,10,11,12]. The N and O in the atmosphere will inevitably enter the weld during TIG welding of Zr-4 alloys, resulting in a significantly higher weld hardness than the base metal (BM) [12]. Coating is another effective protection method for the laser welding of zirconium alloys. A Cr coating with a thickness of 8–10 microns is prepared on the surface of zirconium alloy, and a Cr_2_O_3_ protective layer is generated during the welding process to avoid high-temperature oxidation of the zirconium alloy [13,14,15], but this inevitably reduces the purity of the zirconium alloy weld.

Electron beam welding (EBW) has the advantages of high purity of vacuum-protected weld, high energy density, small heat input and small thermal impact on the BM, which helps to ensure the performance of zirconium alloy welded joints [16,17]. The current research on EBW of zirconium alloys focuses on traditional zirconium alloy, and scholars are more concerned about the corrosion resistance of joints under service conditions. The study by Jha et al. [18] showed that the hardness of Zr-4 alloy electron beam welded joints is higher than that of the BM. The tensile properties of Zr-4 alloy electron beam welded joints are not inferior to those of the parent material [19], but the presence of pores in the weld may affect the corrosion performance of the joint [20]. Bandi et al. [21] demonstrated through Raman spectroscopy that the pores in the Zr-4 electron beam welded joint are mainly hydrogen pores. Zhang et al. [22] found that the EBW joint of Zr-702 alloy is composed of α-Zr and β-Zr, and β-Zr small blocks are distributed along the edges of layered α-Zr, which indicates that the joint has better corrosion resistance than the BM. Yao et al. [23] found that the precipitation of Zr(Fe,Cr)2 s phase particles in the weld seam is the main reason for the increase in hydrogen absorption of the joint after steam corrosion in a high-pressure reactor, and the higher the Cr content in the joint, the greater the hydrogen absorption.

As a new type of zirconium alloy, research on the Zr-Sn-Nb system alloy in relation to welding is still scarce. Scholars have carried out research on dissimilar materials joined by partial transient liquid phase bonding of Zr-Sn-Nb alloy with stainless steel [24] and resistance welding of Zr-Sn-Nb with Zr-Nb alloys [25]. For the welding of a single Zr-Sn-Nb alloy, Wei et al. tested the low-cycle fatigue properties of an electron beam welded Zr-Sn-Nb alloy [26], but research on the microstructure analysis and the performance evaluation of the new Zr-Sn-Nb alloy EBW joints is still insufficient. This article focuses on the evaluation requirements of the service performance of Zr-Sn-Nb alloy welded joints in fuel components. The optimization of Zr-Sn-Nb alloy EBW process parameters was carried out; the formation and microstructure of Zr-Sn-Nb alloy welded joints under optimized parameters were analyzed. The mechanical properties of the joints are tested and evaluated.

## 2. Experimental Section

**Figure 1:**
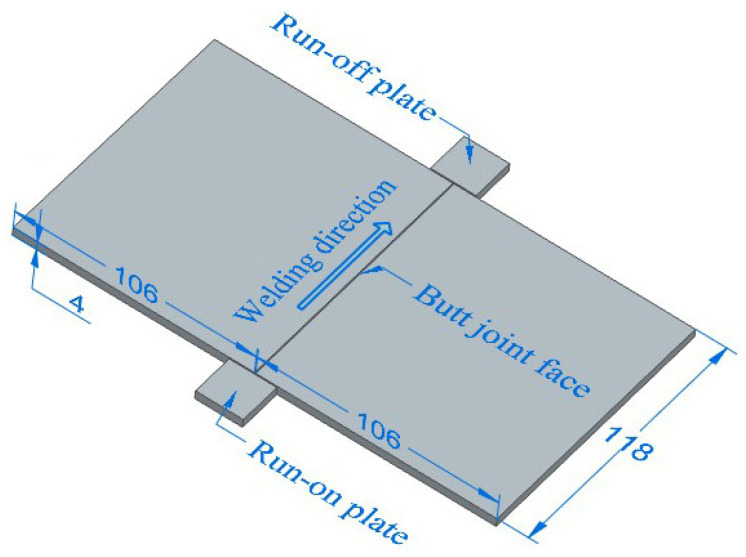
Schematic diagram of EBW assembly of Zr-Sn-Nb plate.

Zr-Sn-Nb alloy plates with a thickness of 4 mm used in this work were cold rolled and then annealed to a fully recrystallized state. The EBM-30H electron beam welder from Kunta was used for the EBW test. Vacuum was drawn to 1.0 × 10^−3^ Pa before welding to prevent the Zr-Sn-Nb alloy from reacting with O and N at high temperature. Two flat plates with dimensions of 118 mm × 106 mm × 4 mm were butt-welded along the length direction (perpendicular to the rolling direction) as shown in Figure 1. After welding and cooling for 10 min, the vacuum chamber was opened to avoid oxidation of the workpiece at high temperatures. Welding process parameters were optimized according to the guidelines of excellent surface formation, backside penetration and low heat input, and the optimized welding process parameters are shown in Table 1. The welding beam current varied from 26 to 28 mA, with 27 mA preferred for weld shaping. After welding, the welded specimens were subjected to vacuum stress relieving and annealing (550 °C, holding time 2 h).

**Figure 2:**
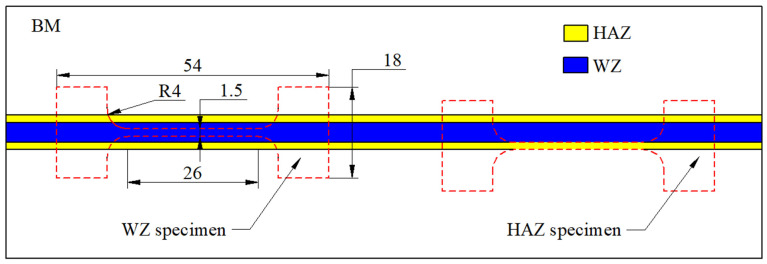
Micro-tensile specimens and dimensions in different areas of the joint.

After welding, wire cutting is used to cut the cross-section of the weld seam to prepare metallographic specimens. After mechanical grounding and polishing, corrosive agents (HF:HNO_3_:H_2_O = 2:9:9) are used for corrosion. X-ray diffractometer (XRD, Panalytical, Empyrean, 8°/min, 20 kV, Cu K_α_) was used to analyze the phase composition of the joint. Olympus-SZX12 metallographic microscope and scanning electron microscope (SEM, Quanta-200 FEG, ThermoFisher, Hillsboro, OR, USA) equipped with energy dispersive spectrometer (EDS, Xplore) were applied to characterize the microstructure of the joint. Electron back-scatter diffraction (EBSD) was carried out to analyze the grain orientation and texture of the joints. The EBSD camera attached to FEI Quanta scanning electron microscope (FEI Company, Hillsboro, OR, USA) and HKL Channel 5 software (Version: 5.11.20405) were used to collect and process the orientation information. The test specimens of welded joints and BM are prepared and tested under the same conditions to analyze the mechanical properties of welded joints relative to the BM. HVS-1000A was used to test the micro-hardness of different areas of the joint, with a loading load of 200 g and a continuous loading time of 10 s. The mechanical properties of the joint were tested on the Shimadzu AG series electronic universal testing machine equipment at room temperature (RT) and 375 °C. The strain rate value for the tensile test was 0.007 (mm/mm)/min. In order to test the tensile properties of the weld zone (WZ) and heat-affected zone (HAZ), the tiny tensile specimen shown in Figure 2 was cut along the welding direction. The width of the parallel section at the center of the specimen was 1.5 mm and the thickness was 2 mm. JBS-300B pendulum impact tester (Shanghai Laiyang Electric Technology Co., Ltd, Shanghai, China) was used to carry out Charpy impact test on V-notched welded joints.

Corrosion test specimens were prepared by intercepting test plates with dimensions of 30 mm × 20 mm × 4 mm, including welds, and the length of the specimen was in the welding direction. The comparison test specimens of BM with the same size were also prepared. The corrosion test conditions were the following: 72 h of corrosion test at 400 °C and 10.3 MPa water vapor.

## 3. Results and Discussion

### 3.1. Weld Forming

**Figure 3:**
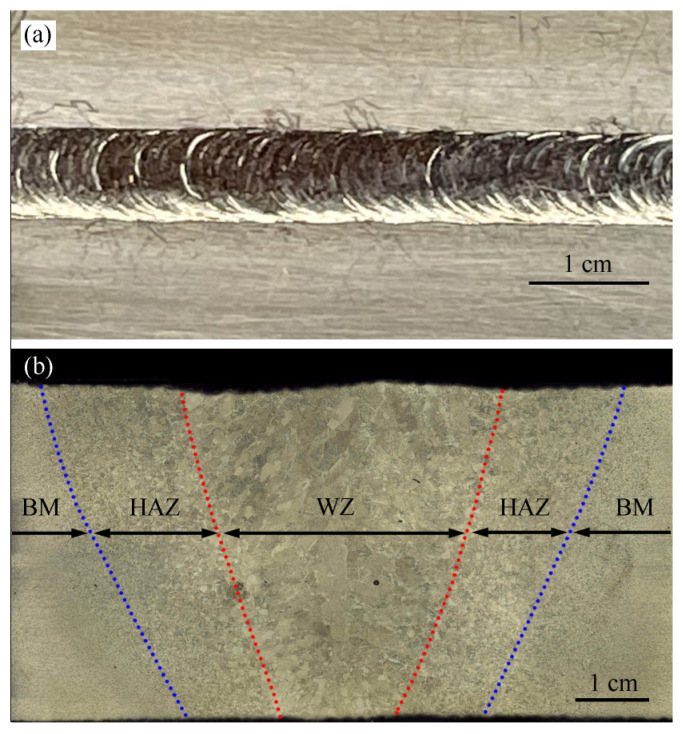
Appearance and cross-section morphologies of Zr-Sn-Nb joint: (**a**) surface appearance; (**b**) cross-sectional morphology.

The morphology of the WZ in the Zr-Sn-Nb joint is shown in Figure 3. The surface of the weld seam is flat, and the fish scale pattern is relatively uniform. No defects such as spatter and undercutting are found, as shown in Figure 3a. From the cross-section of the weld in Figure 3b, it can be seen that the test plate is completely penetrated and slightly adhered to the bottom pad. The widths of the upper part, middle part and root part of the weld are about 4.3 mm, 3.0 mm and 1.5 mm, respectively. And the width of the HAZ on one side of the weld is about 1.7 mm.

### 3.2. Microstructure Analysis

**Figure 4:**
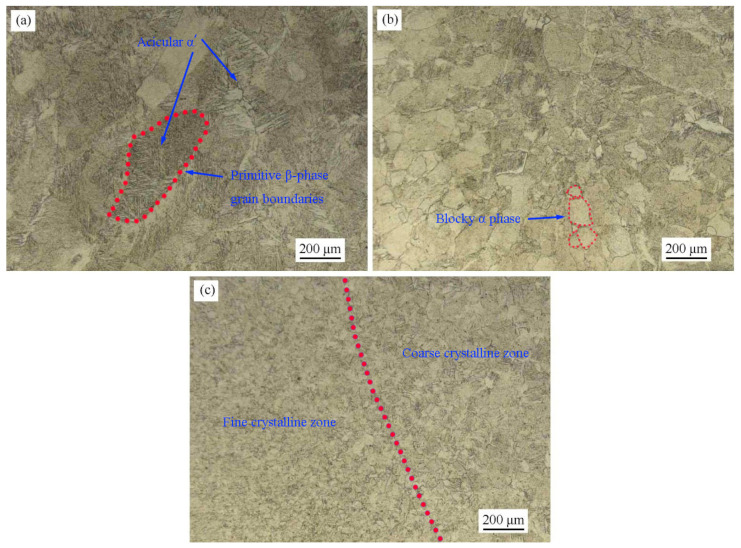
Metallographic microstructure of Zr-Sn-Nb joint: (**a**) upper part of weld; (**b**) lower part of weld; (**c**) HAZ.

Figure 4 shows the metallographic microstructure of different regions of the joint. The grains in the upper region of the weld grow in opposite directions from the fusion line on both sides to the center. The primary β phase generated by solidification transforms into acicular α′ phase during continuous cooling [27]. The coarse primary β-crystal grain boundaries are significant, with the maximum size exceeding 0.5 mm. At the root of the weld, there are coarse equiaxial crystals with a grain size of about 130 μm. The equiaxial crystals are blocky α phase without acicular features. The HAZ is equiaxed in which the grain size is about 80~100 μm near the weld, and the grain size is about 30~40 μm near the BM. During EBW, the molten pool solidified grains grow along the temperature gradient towards the center of the molten pool, resulting in the formation of columnar crystals [28]. The bottom part of the weld is heated relatively less than the top part, the temperature gradient is not significant, and equiaxed crystals are formed under the effect of welding heat. The temperature in HAZ exceeds the recrystallization annealing temperature of the BM, and significant grain growth also occurs in this region. The HAZ close to the weld is severely affected by the welding heat, and the grains in this region are relatively coarse, while the HAZ away from the weld is of fine crystalline.

**Figure 5:**
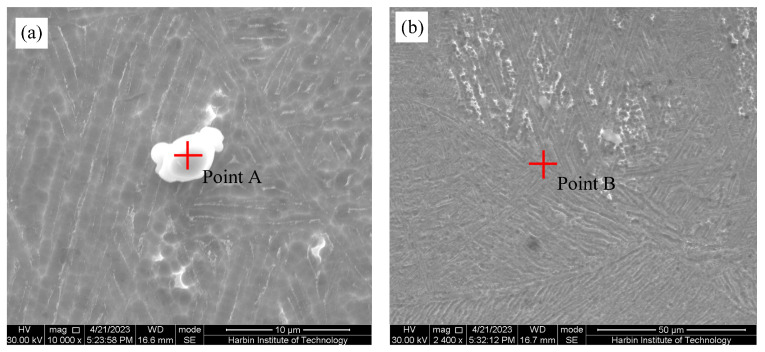
SEM microstructure of WZ: (**a**) white precipitate; (**b**) matrix.

In order to further confirm the microstructure morphology and its compositional characteristics within the weld, SEM-EDS analysis was performed. As shown in Figure 5, WZ contains two main phases, namely the lath-like matrix phase and the second phase precipitated along the grain boundaries. The EDS results of the two phases are shown in Table 2. The precipitated phase (point A) with a size of about 5 μm is dominated by the Nb element. The formation of β-Nb phase is induced when the local Nb/Fe ratio of Zr-Sn-Nb alloy is relatively large. It is assumed that the precipitated phase is the β-Nb phase [29]. The matrix phase is mainly composed of the Zr element, and identified as an α-Zr phase based on the Zr-Nb binary phase diagram [30,31].

### 3.3. XRD Analysis

**Figure 6:**
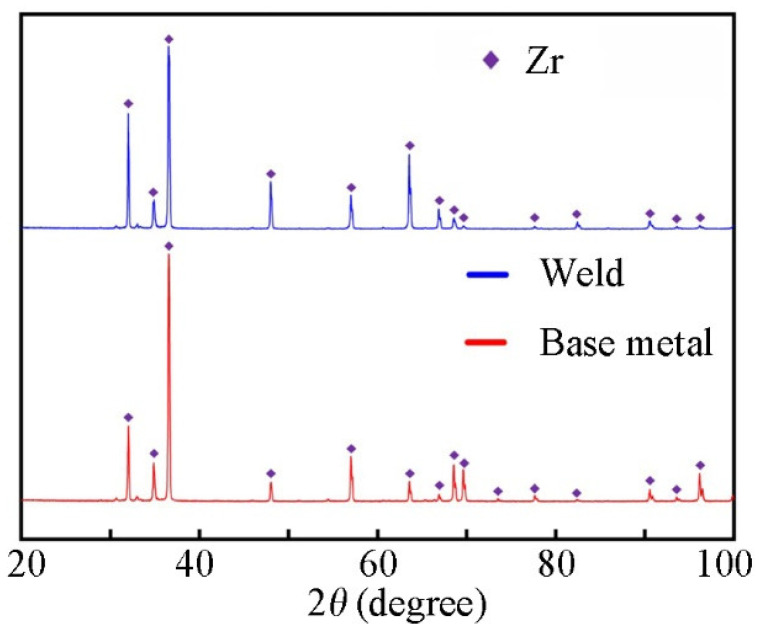
XRD diffraction patterns of BM and WZ.

In order to determine the phases in the BM and WZ, XRD diffraction analysis was performed and the results are shown in Figure 6. From the diffraction pattern, it can be seen that the α-Zr diffraction peak is mainly present in both the BM and WZ, which is consistent with the EDS results in Table 2. The shift in the diffraction peaks of the weld relative to the BM proves the distortion of α-Zr lattice in the weld, which coincides with the observation of acicular α′-Zr in Figure 4.

### 3.4. EBSD Analysis

**Figure 7:**
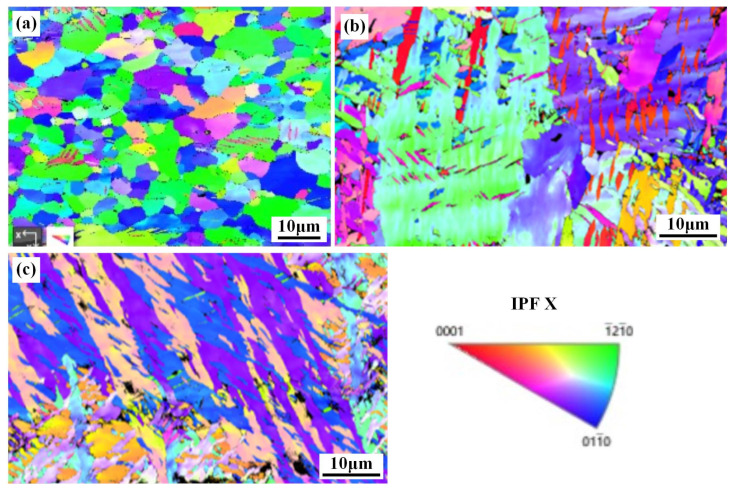
IPF of different regions in the welded joint: (**a**) BM; (**b**) WZ; (**c**) HAZ.

In order to analyze the texture characteristics of different areas in the welded joint, EBSD analysis was conducted. Figure 7 shows the inverse pole figures (IPF) of the microstructure of the BM, WZ and HAZ. Different colors reflect different grain orientations, and blocks with larger color contrasts represent larger grain orientation differences, with red indicating <0001> orientation, green indicating <−12–10> orientation and blue indicating <01–10> orientation. The BM area consists of irregular equiaxed grains, with <−12–10>, <01–10> and <−13–20> grains parallel to the X0 direction being dominant. The grain orientation and microstructure in the HAZ and WZ changed. The grain orientation in the HAZ was relatively uniform, but the orientation of coarse grains <−13–20> and <01–10> were oriented parallel to the X0 direction. The grain orientation in the WZ showed <02–21>, <01–10> and <−12–12> grain direction indices parallel to the X0 direction.

**Figure 8:**
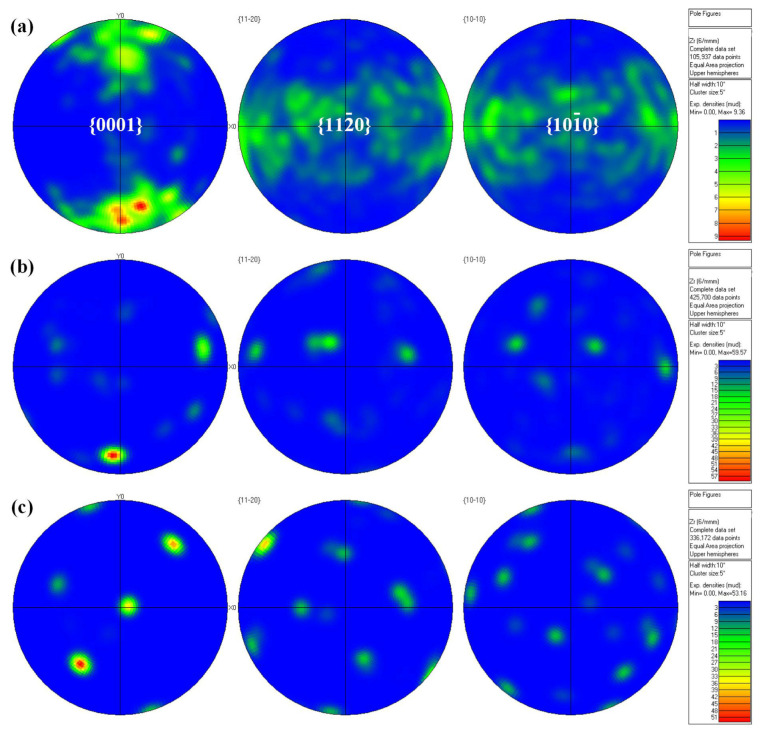
Polar figure of different regions of welded joint: (**a**) BM; (**b**) WZ; (**c**) HAZ.

Figure 8 shows the pole figure of the microstructure in different areas of the joint, where X0 is the rolling direction of the Zr-Sn-Nb alloy flat plate and Y0 is perpendicular to the rolling direction. Contoured pole figures were realized using 10° half width and 5° cluster size. From the pole diagram, it can be seen that BM exhibits a bimodal basal texture, but there is a certain difference in the strength of the texture on both sides. The upper peak is around 6.0 mud in the {0001} pole figure, while the lower peak can reach 9.39 mud, indicating a relatively uniform texture orientation within the BM. The extremely strong points generated in the HAZ and WZ correspond to the BM, but the texture strength of the extremely strong points significantly increases, reaching around 60 mud in strength. Compared with BM, the pole in the HAZ and WZ is more concentrated, indicating that the grain orientation inside the WZ and HAZ is uniform and there is no obvious preferred orientation.

**Figure 9:**
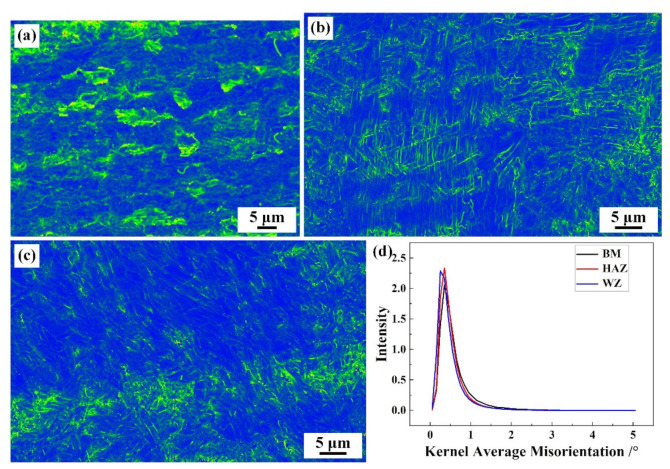
KAM results of different regional microstructures in welded joints: (**a**) BM; (**b**) WZ; (**c**) HAZ; (**d**) Statistics of KAM.

The Kernel Average Misalignment (KAM) analysis results corresponding to different regions within the joint are shown in Figure 9. KAM represents the Kernel-averaged mismatch of the grains, which is the average orientation difference (less than 5°) between a point within the same grain and its nearest neighbor, corresponding to the local orientation gradient induced by deformation within the grain [32,33]. The blue color represents the region with the smallest lattice distortion, with a KAM of 0~1°. The red color represents the region with the highest lattice distortion, with a KAM of 4~5°. From Figure 9, it can be seen that there is a higher content of green areas in the BM, while the green areas in the WZ and HAZ are relatively lower, which is mainly related to their grain size. As the grain size increases, the KAM values of the grains gradually decrease, while the grains in the WZ and HAZ show remarkable coarsening compared to the BM, resulting in a relative decrease in KAM values in the WZ and HAZ. In addition, from the statistics of KAM in different regions in Figure 9d, it can be seen that there is a KAM between 1 and 2° in the WZ and HAZ, which is consistent with the above analysis.

### 3.5. Mechanical Properties Evaluation of Zr-Sn-Nb Joint

#### 3.5.1. Micro-Hardness

**Figure 10:**
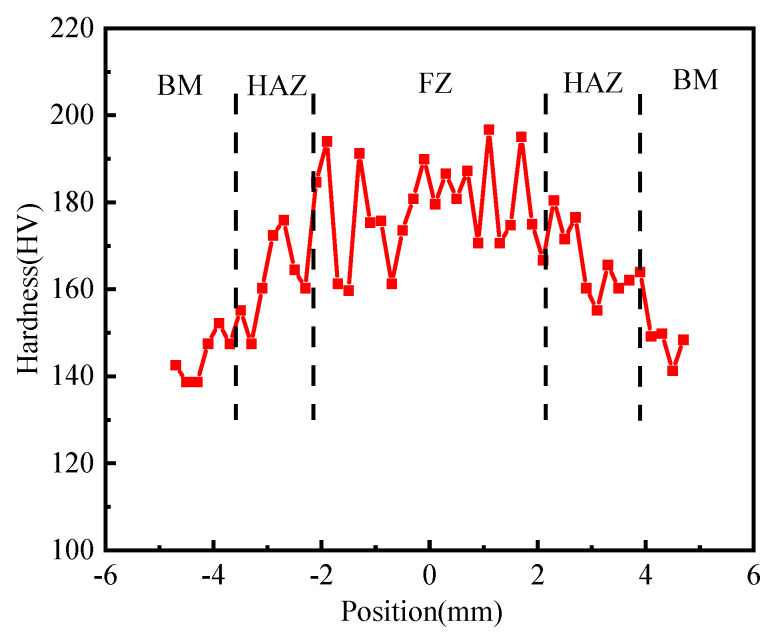
The micro-hardness distribution of Zr-Sn-Nb joint.

Figure 10 shows the micro-hardness distribution curve of the weld cross-section along the vertical weld direction. The micro-hardness of the WZ fluctuates greatly between 160 HV and 194 HV, which is the region with the highest micro-hardness of the joint. The micro-hardness of the HAZ is between 150 HV and 175 HV, and the micro-hardness of the BM is between 141 HV and 159 HV. Welded joints show a trend of gradually decreasing micro-hardness from the WZ to the BM.

#### 3.5.2. Tensile Properties of the Whole Joint

**Figure 11:**
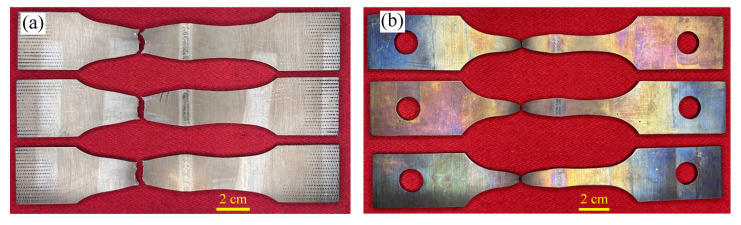
Electron beam welded joints after tensile test: (**a**) RM; (**b**) 375 °C.

**Figure 12:**
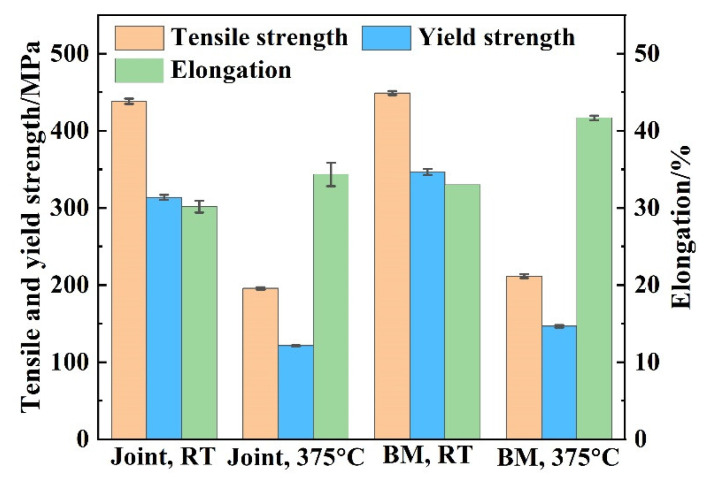
Tensile test results of the joint and BM.

The Zr-Sn-Nb joints were subjected to RT and 375 °C tensile tests and analyzed in comparison with the BM. The specimens after the tensile test of the welded joint are shown in Figure 11. All the specimens fractured in BM. The tensile test results of the joints and BM are shown in Figure 12. The tensile strength, yield strength and elongation of the joint tested at RM were 438 MPa, 313 MPa and about 30.1%, respectively, which reached 97.5%, 90.4% and 91.2% of the BM. On the other hand, the tensile strength, yield strength and elongation of the joint at 375 °C were 195 MPa, 121 MPa and 34.6%, reaching 92.4%, 82.8% and 83.1% of the BM, respectively. After tensile testing, the joints at RT and 375 °C necking occurs on both sides of the weld, and fracture occurs on the side of the BM away from the HAZ. This indicates that the strengths of the WZ and HAZ are higher than that of the BM, and the joint is as strong as the BM. However, the tensile results of the Zr-Sn-Nb joint are slightly lower than the BM, which is related to the fluctuations in the properties of the BM and the error of the test. The yield strength and elongation of the joints at RM and 375 °C are lower than that of the BM, reflecting the decline in the plasticity of the joints.

**Figure 13:**
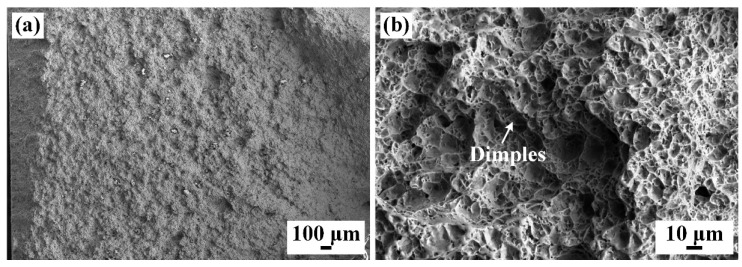
The fracture morphology of Zr-Sn-Nb joint: (**a**) 33×; (**b**) 500×.

Figure 13 shows the fractured surface of the tensile specimen. There are obvious plastic deformation and dimples on the fracture surface, as well as some residual micropores, as shown in Figure 13b. It could be determined that the specimen undergoes the initiation, growth and aggregation of the micropore during the deformation process, ultimately leading to the ductile fracture, which is consistent with the characteristics of micropore aggregation fracture [34].

#### 3.5.3. Tensile Properties of Different Regions of Whole Joint

Due to the extremely narrow weld seam of EBW, the standard tensile test in Section 3.5.2 cannot characterize the performance of the weld seam and HAZ. Therefore, the small tensile specimen shown in Figure 3 is used. The tensile test results of small specimens are shown in Table 3. Compared to tensile tests in Section 3.5.2, the results of small tensile tests on WZ metal show that the average tensile strength of the weld is 499 MPa, 11.6% higher than the BM, and the yield strength is 9% higher than the BM. The average elongation after fracture of the weld and HAZ is 12.5%, only 69.4% of the BM. The results of the small tensile specimen testing confirm that the strengths of the WZ and HAZ are higher than that of the BM, which well explains the joint tensile test fracture at the BM in Figure 11. Coarse grains and fine acicular α′ phases increase the strength of the joint, but reduce the plasticity of the joint [35,36].

#### 3.5.4. Impact Property

**Figure 14:**
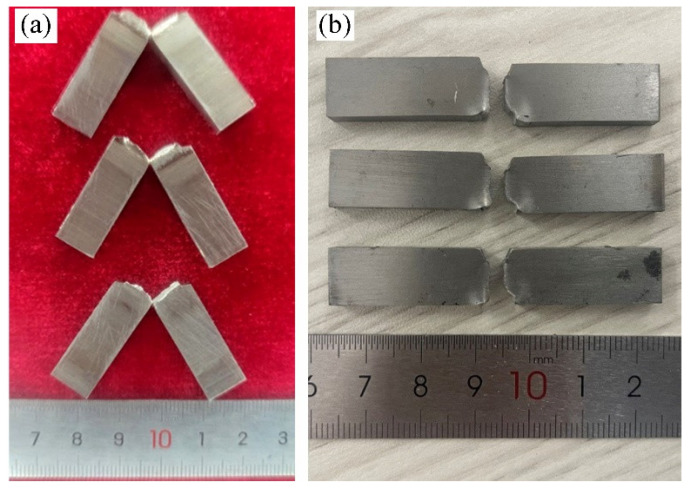
Fracture morphology of specimens: (**a**) joint; (**b**) BM.

Impact property of Zr-Sn-Nb joint and BM were conducted. The fracture morphologies of the specimens are shown in Figure 14. The Zr-Sn-Nb joints were subjected to brittle fracture after impact loading without significantly plastic deformation, two of the specimens were not completely fractured at the root, and the other specimen was completely fractured. The results of impact energy testing are shown in Table 4. The impact performance of the BM is good and has high consistency. The average value of the RM impact energy of welded joints is 18.5 J, which is only 58.3% of that of the BM, indicating that the toughness of the joints decreases significantly relative to that of the BM. This is related to the high hardness of the WZ and HAZ [27].

#### 3.5.5. Corrosion Resistance

**Figure 15:**
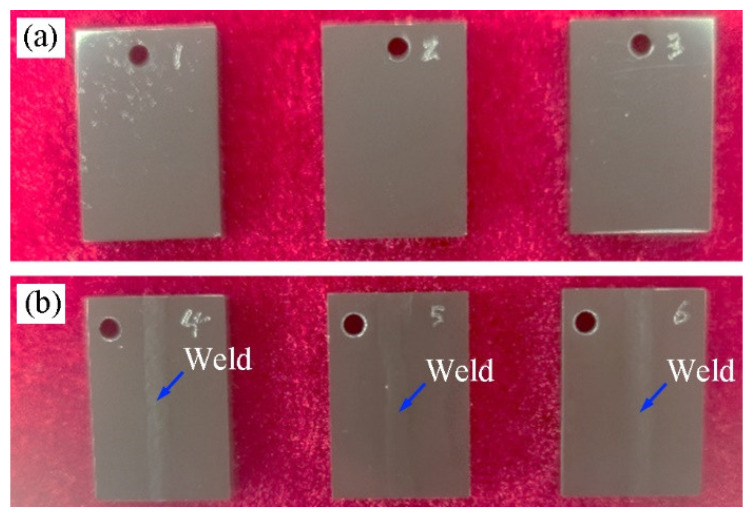
Surface morphology of corroded specimens: (**a**) BM; (**b**) joint.

The specimens of three joints and three specimens of BM after corrosion test are shown in Figure 15. The surface of six specimens changed from the original white gloss to black gloss, and the surface formed a black, dense, glossy and uniform oxidized film. The weld traces can be seen in the center of the joint specimens; the three joint specimens weight gains were 12.35, 12.91 and 13.49 mg/dm^2^, with an average value of 12.91 mg/dm^2^. Three BM specimens’ weight gains were 12.63, 12.63 and 12.66 mg/dm^2^, with an average value of 12.64 mg/dm^2^. It can be seen that the corrosion of the joints’ weight gain average value is higher than that of the BM. The consistency of the test results of the BM is good, while the test values of the joint specimens are relatively scattered.

**Figure 16:**
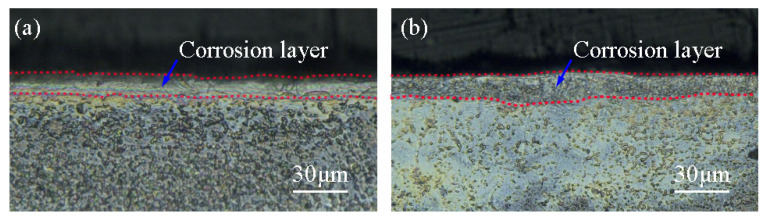
Cross-section morphology of corroded specimens: (**a**) BM; (**b**) joint.

The joint corrosion specimen in the weld is dominated by the BM, and according to the size of the corrosion specimen and the size of the weld in Figure 3, the length of the weld on the cross-section of the corrosion specimen accounts for 12% of the circumference of the cross-section. Assuming that the corrosion rate of the HAZ and the BM is the same, it can be deduced that the corrosion weight gain value at the weld is 14.89 mg/dm^2^, which is 17.8% higher than that of the BM. If the decrease in corrosion resistance in the HAZ is taken into account, the decrease in corrosion resistance of the weld relative to the BM would be even lower. Figure 16 shows the cross-sectional morphology of the corroded specimen. The thickness of the corrosion layer at the BM and WZ varied from 9 to 12 μm and 12 to 15 μm, respectively.

The WZ and HAZ of zirconium have a worse corrosion resistance than the BM in most cases [8]. EBW is carried out in a vacuum environment without the addition of filler metal, which effectively controls the gas contamination from the environment and the variation of weld composition caused by the welding material. On the other hand, there is no significant grain orientation in the joint as shown in Figure 8, which is favorable for the corrosion performance of the joint [37]. Zirconium alloy welded joints have residual stresses, which is an important factor in the reduction in corrosion resistance of the joint [38,39].

## 4. Conclusions

This paper investigated the microstructure and properties of Zr-Sn-Nb alloy EBW joints. The main conclusions are as follows:

(1) EBW of Zr-Sn-Nb alloy can obtain a weld with a flat surface and relatively uniform fish scale pattern. The interior of the weld is free of defects.
(2) The WZ and HAZ grains were significantly coarsened, with the formation of acicular α′-Zr in the upper part of the weld and the maximum size of the primary β grains exceeding 0.5 mm. The blocky α phase formed in the root of the weld. The EBSD results indicate that the grain orientation inside the WZ and HAZ is uniform and there is no obvious preferred orientation.
(3) Coarse grains and fine acicular α′ phases increase the strength of the joint, but reduce the plasticity of the joint. The tensile strengths of the joints at RT and 375 °C were 438 MPa and 313 MPa, respectively, and fracture occurred in the BM. The strength of the WZ and HAZ were higher than that of the BM, but the elongation was significantly lower than that of the BM.
(4) Hardening of the joint significantly reduces the toughness of the joint. The RM impact energy of the joint is 18.5 J, which is only 58.3% of the BM.
(5) The high purity of the EBW process and unsignificant grain orientation minimizes damage to the corrosion resistance of Zr-Sn-Nb alloy joints. The corrosion weight gains of the joint specimen and the BM specimen were 12.91 mg/dm^2^ and 12.64 mg/dm^2^, respectively, and the thicknesses of the cross-section corrosion layer were 12–15 μm and 9–12 μm, respectively.

The significant decrease in joint plasticity and toughness as well as the loss of joint corrosion properties need to be further studied and improved. Post-weld heat treatment is expected to improve the welded joint properties of Zr-Sn-Nb alloys. This will be carried out in subsequent work.

## Figures and Tables

**Table 1:** EBW parameters of Zr-Sn-Nb alloy.

Acceleration Voltage /kV	Focus Current /mA	Welding Beam Current /mA	Welding Speed /(mm·min^−1^)	Working Distance /mm
60	714 *	26–28	480	190.5

* The focused electron beam spot is about 8 mm higher than the surface of the workpiece.

**Table 2:** EDS results of WZ in Figure 5 (at.%).

Point Position	Sn	Cr	Fe	Zr	Nb	Possible Phases
A	0.89	1.22	1.29	18.12	78.48	β-Nb
B	0.8	0	0	99.2	0	α-Zr

**Table 3:** Tensile test results of different regions of Zr-Sn-Nb alloy welded joints.

	WZ	HAZ	BM
Tensile strength (MPa)	499	496	447
Yield strength (MPa)	368	386	337
Elongation (%)	12.5	12.5	18.0

**Table 4:** Impact energy of Zr-Sn-Nb joints (J).

	Sample 1	Sample 2	Sample 3	Average Value
WZ	20.5	17	18	18.5
BM	31.3	31.5	32.3	31.7

## Data Availability

The raw data generated during the present study are available from the corresponding author on reasonable request.
